# Genome-Wide Transcriptional Profiling of *Clostridium perfringens* SM101 during Sporulation Extends the Core of Putative Sporulation Genes and Genes Determining Spore Properties and Germination Characteristics

**DOI:** 10.1371/journal.pone.0127036

**Published:** 2015-05-15

**Authors:** Yinghua Xiao, Sacha A. F. T. van Hijum, Tjakko Abee, Marjon H. J. Wells-Bennik

**Affiliations:** 1 NIZO food research, Ede, The Netherlands; 2 Top Institute Food and Nutrition, Wageningen, The Netherlands; 3 Center for Molecular and Biomolecular Informatics, Radboud University Medical Center, Nijmegen, The Netherlands; 4 Laboratory of Food Microbiology, Wageningen University, Wageningen, The Netherlands; Beijing Institute of Microbiology and Epidemiology, CHINA

## Abstract

The formation of bacterial spores is a highly regulated process and the ultimate properties of the spores are determined during sporulation and subsequent maturation. A wide variety of genes that are expressed during sporulation determine spore properties such as resistance to heat and other adverse environmental conditions, dormancy and germination responses. In this study we characterized the sporulation phases of *C*. *perfringens* enterotoxic strain SM101 based on morphological characteristics, biomass accumulation (OD_600_), the total viable counts of cells plus spores, the viable count of heat resistant spores alone, the pH of the supernatant, enterotoxin production and dipicolinic acid accumulation. Subsequently, whole-genome expression profiling during key phases of the sporulation process was performed using DNA microarrays, and genes were clustered based on their time-course expression profiles during sporulation. The majority of previously characterized *C*. *perfringens* germination genes showed upregulated expression profiles in time during sporulation and belonged to two main clusters of genes. These clusters with up-regulated genes contained a large number of *C*. *perfringens* genes which are homologs of *Bacillus* genes with roles in sporulation and germination; this study therefore suggests that those homologs are functional in *C*. *perfringens*. A comprehensive homology search revealed that approximately half of the upregulated genes in the two clusters are conserved within a broad range of sporeforming Firmicutes. Another 30% of upregulated genes in the two clusters were found only in *Clostridium* species, while the remaining 20% appeared to be specific for *C*. *perfringens*. These newly identified genes may add to the repertoire of genes with roles in sporulation and determining spore properties including germination behavior. Their exact roles remain to be elucidated in future studies.

## Introduction

*Clostridium perfringens* is the causative agent of various animal and human diseases including clinical gas gangrene and foodborne diarrhea [1]. Its dormant endospores are highly resistant to environmental insults, such as heat, draught, sanitizing agents, and preservatives. This allows for the widespread occurrence of this anaerobe in food materials and in the intestinal tract of humans and animals [2]. For *Bacillus* and *Clostridium* species, the process of spore formation, called sporulation, starts with asymmetric cell division and is characterized by different stages in which a forespore is formed and engulfed, followed by formation of a cortex layer and completion of a spore coat layer [3]. Noteworthy is that certain *C*. *perfringens* strains produce and release diarrhea-causing enterotoxin (CPE) during sporulation. The production of this toxin is strictly associated with spore formation and mother cell lysis in the gastrointestinal tract [4].

Morphological changes of sporulating cells and genes involved in the sporulation cascade have been intensively studied in *Bacillus* species, particularly in *B*. *subtilis* [5]. For clostridia, these phenomena have been investigated for the industrially relevant *C*. *acetobutylicum* and clinically relevant *C*. *difficile* [6,7]. So far, detailed studies on global gene expression during sporulation have not been reported for *C*. *perfringens*. The sporulation cascades of bacilli and clostridia share many common features, but also have distinct features, with one of the major differences being indirect Spo0A phosphorylation in bacilli versus direct Spo0A phosphorylation in clostridia during the initiation stage of sporulation [3,8–10].

Bacterial spores can germinate under favorable conditions. This process is characterized by a transition from phase bright to phase dark under a phase contrast microscope and is associated with the release of the major spore core component Ca^2+^-dipicolinic acid (DPA) [11]. Germination is irreversible and upon germination the spore loses its resistance to heat and other treatments. Full germination furthermore requires coat and cortex degradation, upon which a vegetative cell can emerge and return to vegetative growth [12]. For *C*. *perfringens*, growth under optimal conditions is fastidious. In the case of foodborne illness, the pathogen causes disease at relatively high numbers (>10^5^) of vegetative cells, associated with sporulation in the human gut [13,14]. To control *C*. *perfringens* in foods, different strategies can be envisioned, including complete prevention of germination and outgrowth, or alternatively complete germination in combination with an inactivation treatment that kills the sensitive germinated spores. However, the efficacy of such approaches may be affected by diversity in germination behavior between species and strains, and heterogeneity in germination [15–17].

Various germination-related genes of *C*. *perfringens* have previously been identified. These include the *gerK* locus, which encodes a variant of the classical *B*. *subtilis* tricistronic *gerA* operon, in which GerKC plays an essential role in the sensing of germinants [18–21]. The presence of *prkC*, encoding a Ser/Thr kinase in *C*. *perfringens*, suggests the existence of an alternative germination pathway triggered by environmental peptidoglycan fragments, as described for *B*. *subtilis* [20,22–24]. *C*. *perfringens* also carries the gene *gpr*, encoding a germination specific protease which plays an essential role in the degradation of small acid-soluble proteins (SASPs) that are known to stabilize and protect *B*. *subtilis* spore DNA from lethal damages [25]. Cortex degradation of *C*. *perfringens* has been shown to require a spore cortex-lytic enzyme encoded by *sleC*, a cortical fragment-lytic enzyme encoded by *sleM*, and serine proteases encoded by *csp* genes. Despite *in vitro* evidence suggesting that SleM degrades cortical fragments, functional analysis in *C*. *difficile* and *C*. *perfringens* has demonstrated that the serine protease CspB and SleC are essential for spore germination [26–29].

*In silico* analysis suggested that this mechanism is present in *C*. *perfringens*, *C*. *difficile*, *C*. *tetani*, *C*. *beijerinckii* and specific strains of *C*. *botulinum* [20,24]. The expression of the majority of genes that encode characterized germination proteins, including *gerKA*, *gerKB*, *gerKC*, *sleC*, *sleM*, and *cspB*, is highly sporulation-specific [19,21,26,27,30].

Recently, Galperin and colleagues compiled a list of all reported sporulation genes in a wide range of species [8]. That list provides an excellent foundation to identify homologous genes in *C*. *perfringens* with putative roles in sporulation [8]. Actual experimental data on the expression of such homologs in *C*. *perfringens* during sporulation would substantiate their role in the sporulation process of this bacterium.

In the current study, we characterized the sporulation phases of *C*. *perfringens* SM101 by assessing changes in morphology, biomass accumulation (OD_600_), the total viable counts of cells plus spores, the viable count of heat resistant spores alone, the pH of the supernatant, CPE production, and DPA accumulation. So far, strain SM101 is the only publicly available chromosomal-*cpe* strain with a completed genome sequence [31] and this strain has been the subject of various studies on sporulation and spore germination [19,21,26,27,32,33]. Whole-genome microarray analysis was performed throughout a time course during sporulation. This confirmed expression of many known and predicted sporulation and germination genes in *C*. *perfringens*. Moreover, it revealed novel putative sporulation genes that were specifically up-regulated during sporulation and conserved amongst bacilli and clostridia. Such genes are likely candidates to play a role in sporulation and/or spore properties.

## Materials and Methods

### Bacterial strain, growth conditions and sampling

*C*. *perfringens* SM101 (kindly provided by Dr. Melville, Blacksburg, VA, US) was stored in Cooked Meat Broth (BD, Sparks, US) at 1°C and used as a working stock. Sporulation was performed as previously described in 500 ml modified Duncan-Strong (mDS) sporulating medium [17,34]. In short, 50 μl aliquots of the stock culture were inoculated in 10 ml pre-reduced Fluid Thioglycollate Medium (FTM, BD, Sparks, USA), heated at 75°C for 15 min, and subsequently incubated anaerobically at 37°C overnight in an anaerobic chamber (gas mixture: 10% H_2_, 5% CO_2_, 85% N_2_, v:v:v). An exponential-phase inoculum was prepared by mixing 1 ml of the overnight culture and 9 ml FTM followed by 4 h incubation; the fast dividing vegetative cells produced substantial amounts of gas. An inoculum (1:10,000, v:v) was added to 500 ml of pre-reduced mDS medium and incubated at 37°C. This sporulating culture was continuously mixed using a magnetic stirrer. Samples of the sporulating culture were taken every 30 min starting 2 h after inoculation. Twenty-five-milliliter aliquots of the culture were collected, of which 1 ml was used to determine the total viable counts (vegetative cells plus spores) and the remaining 24 ml was taken out of the chamber to assess biomass accumulation (OD_600_), the total viable counts of cells plus spores, the viable count of heat resistant spores alone, the pH of the supernatant, CPE production and DPA accumulation (see below).

### Analyses during sporulation

**Fig 1:**
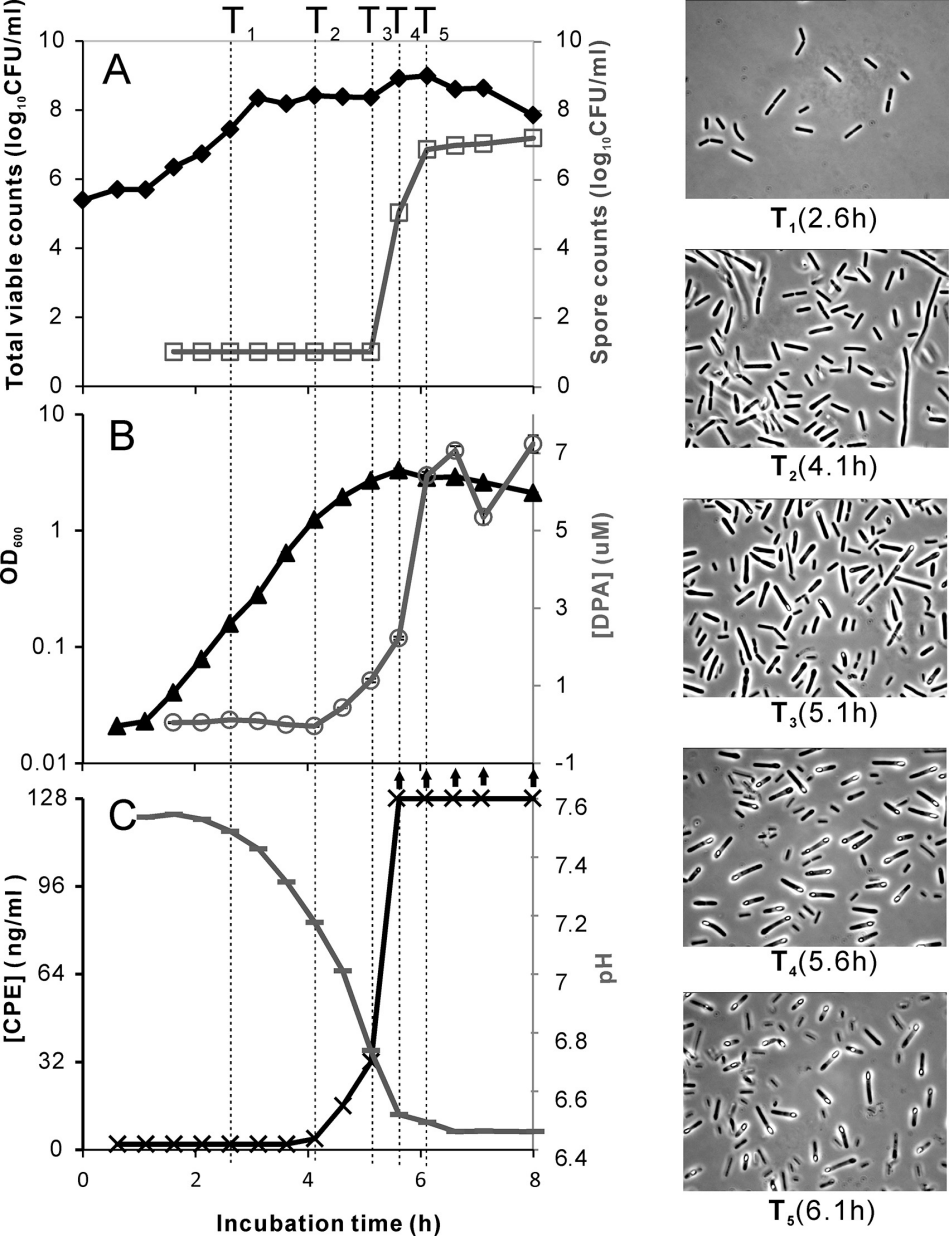
Analysis of a sporulating culture of *C*. *perfringens* SM101. In the culture growing in mDS, the following parameters were determined in time: biomass accumulation (OD_600_), the total viable counts of cells plus spores, the viable count of heat resistant spores alone, the pH of the supernatant, CPE production and DPA accumulation. Phase contract microscopic images were taken at representative time points of five phases (magnification: 1000×). Fig 1A shows total viable counts (♦) and spore counts (□); B shows OD_600_ (▲) and DPA accumulation in forespores/spores (○); C shows pH (-) and CPE in supernatant (**×**)*. * The detection range of the PET-RPLA was 2 to 128 ng/ml. Samples with CPE concentrations exceeding 128 ng/ml are indicated with an arrow pointing upwards.

The following indicators of biological events were assessed to determine phases of sporulation using the 24 ml sample mentioned above (see also Fig 1).

Biomass accumulation was determined by measuring the optical density at 600 nm (OD_600_) using an Ultraspec 3100 pro spectrophotometer (Amersham Bioscience, Freiburg, Germany).

Total viable counts of *C*. *perfringens* were determined by plating serial dilutions on BHI agar plates (BD, Sparks, US) in the anaerobic chamber. Spore counts were determined by first heating the sample to 75°C for 10 min, followed by plating on BHI plates. Colonies were counted after 24 h of anaerobic incubation.

The culture pH and CPE release were measured as follows: a 5 ml culture was subjected to centrifugation (16,000 ×g for 5 min) at 4°C. The supernatant and the pellet, which contained the cells and spores, were separated. The supernatant was further filter-sterilized using 0.2 syringe filters (Whatman, Germany). The pH in the filter-sterilized supernatant was measured using a pH meter (WTW, Weilheim Germany). The CPE concentration was measured using the PET-RPLA kit (Oxoid, Hampshire, UK). This assay is designed to detect CPE in a range of 2 to 128 ng/ml according to the manufacturer. Besides measuring CPE in the supernatant, it was also assessed whether CPE had accumulated in sporulating cells. To this end, half of the pellets were disrupted using Lysing Matrix B (0.1 mm silica spheres, MP Biomedicals) and re-suspended in 2.5 ml supernatant, followed by CPE measurement using the PET-RPLA test.

DPA accumulation was measured using the other half of the harvested pellet, which was washed in phosphate buffered saline (PBS, containing 130 mM NaCl, 10 mM sodium phosphate, pH 7.4) and autoclaved at 121°C for 15 min to release DPA from forespores and spores. DPA concentrations were determined by measuring the emission at 545 nm of the fluorescent terbium-DPA complex in a Safire II plate reader equipped with a B122253 fluorescence top module (TECAN, Austria) as previously described [35].

Furthermore, samples were examined microscopically. Fixation and microscopic imaging was performed as follows. Harvested cells and/or spores originating from 5 ml of a culture were fixed directly upon removal from the anaerobic chamber using 3 ml cold 4% paraformaldehyde (Sigma, Steinheim, Germany) solution followed by incubation at 4°C for 16 h. The fixed samples were washed once with a mixture of PBS and 0.1% Tergitol-type NP-40 (Sigma-Aldrich, St. Louis, MO, US) (9:1, v:v), and once with PBS only. The pellet was then resuspended in a cold mixture (1:1, v:v) of PBS and ethanol (99.9%). Aliquots of 100 μl were made and stored at -20°C for at least 1 h. Complete fixation of spores was confirmed by spreading 0.1 ml fixed *C*. *perfringens* spore suspension on BHI agar. No colony formation was observed on the plates after anaerobic incubation at 37°C for 3 days. The morphological phases of sporulation were examined for all samples using phase-contract microscopy (magnification 1000×) (see Fig 1). The microscopic images were collected using an imaging system (Zeiss Axioplan microscope with Zeiss HRc camera).

### Preparation of total RNA samples

Total RNA was extracted from cells and spores throughout the sporulation process in mDS. First, a 3 ml mixture of acidified phenol-ethanol (1:9, v:v) was added to 12 ml culture samples immediately upon sampling. After brief mixing using a vortex, this sample was kept on ice for 30 min. Cells/spores were then harvested by centrifugation at 16,000×g for 5 min at 4°C, and resuspended in 1 ml ice cold TRIzol Reagent (Ambion, US). This procedure was used to minimize the effects of exposure to *e*.*g*. oxygen, chilling and centrifugation on transcripts and to prevent RNA degradation. Aliquots of 0.6 ml cells/spores in trizol mixture were transferred to Lysing Matrix B tubes prefilled with 0.1 mm silica spheres. These samples were frozen in liquid N_2_ and kept at -80°C until use.

For final RNA extraction, 0.4 ml ice-cold TRIzol Reagent was added to the frozen sample and homogenization was performed immediately—without thawing—using Savant FastPrep FP120 (Qbiogene, Carlsbad, US) for 40 s at Speed 6, in total 3 times with interval cooling on ice. After phase separation, RNA precipitation and washing was performed as described in the instructions of the manufacturer. The total RNA extracts were further purified using an RNeasy kit (QIAGEN, Hilgen, Germany). On-column DNA digestion was performed during purification using RNase-Free DNase (QIAGEN, Hilgen, Germany). RNA concentrations were determined using a Nanodrop analyzer (Thermo Fisher Scientific, Wilmington, US) and RNA was confirmed to be intact using a Bioanalyzer (Agilent technologies, Palo Alto, CA, US).

### RT-PCR

**Fig 2:**
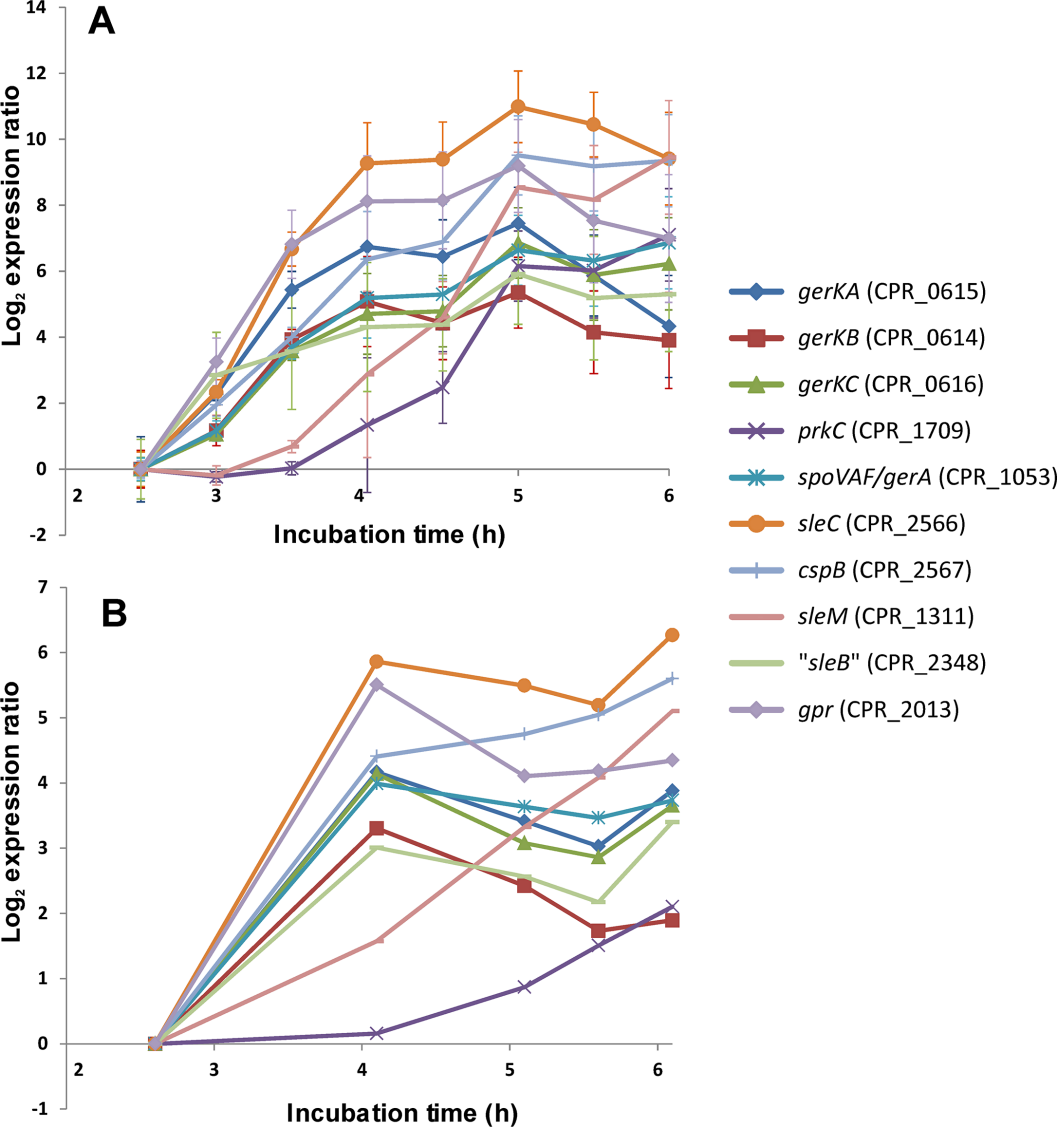
Time course expression of germination-related genes assessed by RT-PCR (A) and microarray analysis (B). Samples were taken from *C*. *perfringens* cultures growing in mDS (A) every 0.5 h after inoculation. Transcripts of genes *dnaE* and *sigH* were used to normalize *Ct* values resulting from RT-PCR. RNA from culture samples taken at the five representative time points was used for microarray expression profiling (B). The data extraction and presentation is described in the Materials and Methods section.

In our previous genome-mining study, various germination genes were predicted to play essential roles in germination of clostridia (e.g. homologs of *B*. *subtilis* germination genes) [24]. RT-PCR was employed prior to whole-genome expression profiling to verify expression of a number of genes that are known to be involved in germination and which are expressed during sporulation. Therefore, samples were taken from two independent cultures every half hour throughout growth and sporulation. For each sample, RNA was isolated (see above). Purified total RNA of each sample was then subjected to reverse transcription in duplicate using the SuperScript III First-Strand Synthesis System for RT-PCR (Invitrogen, Paisley, UK) according to the instructions of the manufacturer. The generated cDNA was subsequently amplified by real-time PCR using the primer pairs listed in Table 1 for the corresponding targets. Serial dilutions of a genomic DNA sample of strain SM101 were prepared as described before [17] and this was used as a control in this multiple-plate application. Transcripts of genes *dnaE* and *sigH* [6,36] were used to normalize the resulting *Ct* values. Thus, for two independent cultures, duplicate RT-PCR results were obtained per time point. The average expression per time point for these two cultures was calculated and is shown in Fig 2A.

**Table 1:** Primers for RT-PCR used in this study.

Locus tag	Gene	Primer	Sequence (5’-3’)	Product size (bp)	Reference
CPR_1053	*spoVAF (gerA)*	gerA-403F	AGGGGTTCTAGGGATGGCTTTGT	236	This study
		gerA-644R	ACAAGGCTTTGTTCGCTCATGGT		[21]
CPR_0615	*gerKA*	gerKA-F1	GTATAGGGAGGTGGATACAG	192	This study
		gerKA-190sR	TCGTTTTTATCCTTTGACCAATTT		
CPR_0614	*gerKB*	gerKB-006F	TTTGGGAAAGCTAAATACAAGACA	112	This study
		gerKB-118R	TCCAAGTATCTCTTCCGCCTA		
CPR_0616	*gerKC*	gerKC-622F	TTAAGCGGAGGAGCTTTGTT	128	This study
		gerKC-752R	GGGTCTTGAGGGTTCATAACTTC		
CPR_1709	*prkC*	prkC-1182F	TTGGAACAACAACTGGAGACA	167	This study
		prkC-836-426sR	AATAATGAAAGCTATGGAAAAGGA		
CPR_2566	*sleC*	sleC-698F	CGGCTTTTGATCATGCTTTT	170	This study
		sleC-868R	CCCATTGAGTCATCCAACCT		
CPR_2348	CPR_2348	CPR2348-423F	TGACGTAGTCCCTGATGGTGATAGC	127	This study
		CPR2348-550R	CGACGCCTTTCATCCACGAGCA		
CPR_1311	*sleM*	sleM-638F	ATTTAAACTGGGGGCCAAAT	233	This study
		sleM-871R	TTGGCTTTCCTTTTGGAAGA		
CPR_2013	*gpr*	gpr-295F	ACGGCTTTAGTAGTTGGGCTTGGA	165	This study
		gpr-460R	TGCCTAAAACTCCAGGTGCT		
CPR_2418	*sigH*	sigH-195F	TCAAGAGGGAATGATTGGATT	134	This study
		sigH-329R	TTTTGCCTTGTTGCAGTTTTT		
CPR_0339	*dnaE*	dnaE-1F	TCATCAACTCACGCTGCGGGA	128	This study
		dnaE-1R	TCCACAGCATCACGCATAACAGTT		
CPR_2567	*cspB*	cspB-1368F	TGGTAGGGGCGTTGTTAGAC	152	This study
		cspB-1520R	AGAAGAGCGCATATCCCAGA		

### Time-course genome expression profiling and clustering

#### Probe design

Microarrays were obtained from Agilent technologies (Santa Clara, CA, US). Probes were specified for ORFs that were present in the genomes of *C*. *perfringens* SM101. Probes were designed separately for the plasmid and chromosomal ORFs. The chromosomal ORFs were aligned with BLAST [37] and chromosomal ORFs that were (virtually) identical were grouped based on a 95% or higher nucleotide identity over the full length of the ORF. The virtually identical ORFs were aligned by Clustal W v2.0.1 [38], and the identical regions (which are most suited for probe design) were selected as consensus ORF fragments using an in-house developed script. Probe design was performed on the plasmid ORFs, the unique chromosomal ORFs and the consensus fragments of near-identical ORFs, resulting in about 5 probes per ORF. All probes were designed with an aim length of 60 bp (with a minimum of 55 bp) and a minimum probe distance of 100 bp using OligoWiz v2.1.3 [39] using default parameters for prokaryotic long-mers. The total probe score was based on weighting the individual scores for (i) cross-hybridization: 39.0%, (ii) delta Tm: 26.0%, (iii) folding: 13.0%, (iv) position: 13.0%, and (v) low complexity: 9.1%. This custom probe design contained 26,876 probes (GPL 18980), which were spotted on 4×44K probe glass slides. In total, 96.7% (2532/2619) of protein-coding ORFs of strain SM101 were represented on the microarray by at least one probe.

#### Experimental design, labeling and hybridization

Based on analyses of the *C*. *perfringens* SM101 cultures throughout growth and sporulation, five time points were selected that represented different phases of the spore-forming procedure, namely, exponential growth (T_1_), early stationary phase (T_2_), early sporulation (T_3_), late sporulation (T_4_) and cell lysis (T_5_). These different phases were assessed in two independent experiments, A and B, performed on different days. The exact time between inoculation and the typical events in the different phases was not identical between the duplicates. For experiment A, the five sampling times, which are typical for the five observed phases, namely, T_1_ to T_5_, were at 2.2, 3.5, 4.5, 5.0, and 6.0 h after inoculation, respectively. For experiment B, the sampling times representing the five typical phases were at 2.6, 4.1, 5.1, 5.6 and 6.1 h after inoculation, respectively.

For each sample taken at a certain time point, 3 μg of total RNA was subjected to cDNA synthesis using random nonamer priming. Each sample was labeled with either Cy3 or Cy5 using a Cyscribe postlabeling kit (Amersham Biosciences, UK) according to the protocol of the manufacturer. A more detailed description was published previously [40]. A scheme was designed that allowed for hybridization of each sample in duplicate or triplicate, at the same time allowing for normalization of expression data from the two independent experiments A and B. This loop design is presented in S1 Fig. The subsequent steps in the microarray expression profiling experiments included concentration of labeled cDNA mixtures, hybridization, washing, slide scanning, spot quantification and normalization. This was performed as described previously [41], with adjustments to reaction volumes according to instructions of the manufacturer for 44K arrays. The microarray data have been deposited in NCBI's Gene Expression Omnibus (GSE59616).

#### Clustering

**Fig 3:**
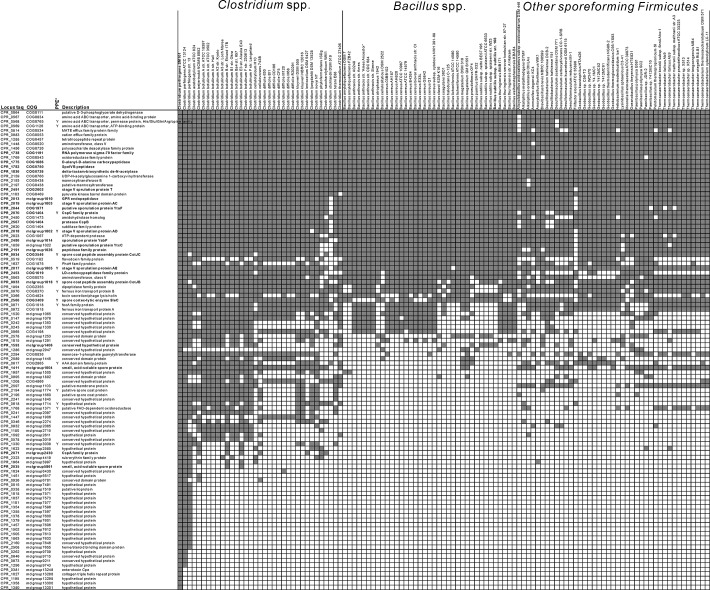
Cluster 1 genes and the occurrence of their homologs in other sporeforming Firmicutes. The genes in Cluster 1 are highly up-regulated during *C*. *perfringens* sporulation. The genes with known sporulation-related functions are indicated in bold against a grey background; the ones with putative functions (poorly characterized and uncharacterized) have a grey background only, while the others without specific highlights have not been associated with sporulation or are not known to determine spore properties. PPE is an abbreviation for the Potential of Polar Effect, as co-expression may occur due to high levels of expression of upstream genes in the same operon.

The expression levels of genes during the different sporulation phases at time points T_2_ to T_5_ were compared with those at T_1_ (exponential growth phase). The transcription profiles of all genes throughout the sporulation process were plotted and genes with similar transcription profiles during the time course were clustered with a *k*-means cluster analysis using Euclidean distance,50 maximum iterations and 6 predefined clusters (Genesis, v1.7.6) [42]. The time-course expression profiles of the two independently performed experiments were subjected to the clustering (see Fig 3). Sporulation-specific upregulated genes were grouped in two clusters, named Clusters 1 and 2, and genes belonging to these two clusters were the focus of further analyses as described below.

### Homolog search in sporeforming Firmicutes

The occurrence of homologs of *C*. *perfringens* genes with increased expression during sporulation (belonging to Cluster 1 and 2) was analyzed in a wide range of endospore-forming Firmicutes; genes present in 113 publicly available genome sequences of strains belonging to the Firmicutes were evaluated. Briefly, the chromosomal and protein coding sequences of these strains were obtained from the National Center for Biotechnology Information (NCBI) site. The genes were first assigned to Clusters of Orthologous Groups of proteins (COGs) [43] using KOGNITOR [44]. The remaining genes were clustered using OrthoMCL with default parameters [45] resulting in orthologous groups of genes (OGs). COGs are more generic and the additional OGs likely signify more specific gene functions.

Predicted operons in the genome of *C*. *perfringens* SM101 were obtained from two independent sources using different methods [46,47]. MGcV [48] was used to visualize all genes of Cluster 1 and 2 together with the transcriptional terminators predicted using TransTerm [49]. The genes with potential co-expression are labeled in Fig 3 for Cluster 1 and S2 Table for Cluster 2.

## Results

### Five phases of growth and sporulation were observed for *C*. *perfringens*, based on morphological characteristics and other parameters, including biomass, viable counts of cells and spores, culture pH, CPE production and DPA accumulation

To select time points during sporulation that are representative for the different phases of *C*. *perfringens* sporulation, microscopic analyses was performed to assess morphological changes during the fast sporulation process of this bacterium. At the same time, biomass accumulation (OD_600_), the total viable counts of cells plus spores, the viable count of heat resistant spores alone, the pH of the supernatant, CPE production and DPA accumulation were measured to determine key events during spore formation. These data are presented in Fig 1. Based on the biological events, five phases could be distinguished:

Phase **I** consisted of exponential growth (Fig 1A). Starting from an inoculum of 1×10^5^ CFU/ml, the culture reached a level of 1×10^8^ CFU/ml in approximately 3 h. This phase of exponential growth was characterized by an increase in total viable counts with a calculated generation time of 11 min, and biomass accumulation to an OD_600_ of 0.3. During this phase, spores were not formed, DPA and CPE were not detected and the culture pH remained high at around 7.5. Microscopic analysis showed actively dividing cells at relatively low densities. A representative time point is indicated by T_1_.

During Phase **II,** the total viable counts remained constant (Fig 1A) while the OD_600_ continued to increase (Fig 1B). During this phase, no bright-phase spores were observed (T2 in Fig 1). DPA and CPE were not detectable and the pH of the culture showed a slight decrease to approximately 7.2. A representative time point of this phase is indicated by T_2_.

During Phase **III**, the total viable counts and the OD_600_ were stable and at their maximum level. Microscopic analysis clearly showed fore-spores that were formed in the mother cells, with a small proportion of the spores appearing phase bright. In this phase, the spores were not heat resistant. DPA levels were increasing (corresponding with spore formation) and CPE was detectable with increasing levels during this phase. The pH of the culture decreased further to approximately 6.7. T_3_ shows a representative time point of Phase III.

Phase **IV** was characterized by the occurrence of heat resistant spores; within a timeframe of about one hour, the level of heat resistant spores increased from undetectable to approximately 1×10^7^ CFU/ml, *i*.*e*. the maximum level. During this phase, DPA increased rapidly to the maximum level. The concentration of CPE in the culture exceeded 128 ng/ml. Microscopic analysis revealed that the majority of cells consisted of mother cells carrying phase bright spores. Time T_4_ is a representative point in the middle of Phase IV.

Phase **V** was characterized by a stable concentration of heat resistant spores and DPA, indicative of completion of the spore formation process, albeit that the majority of the spores were still retained in the mother cells. The total viable counts started to decrease and approached the level of spore counts. Time point T_5_ is a very early point in this phase, and was selected as a sampling point to minimize the effect of cellular RNAse released by lysing mother cells.

### Sporulation-specific gene expression patterns are shared by known germination-related genes

Expression of 12 known germination related genes of *C*. *perfringens* SM101 was assessed throughout growth and sporulation in mDS sporulation medium, namely *spoVAF (gerA)*, *gerKA*, *gerKB*, *gerKC*, *prkC*, *sleC*, CPR_2348, *sleM*, *gpr*, *sigHi dnaE*, and *cspB* (Fig 2A). As a pilot experiment, and to test the quality of the isolated RNA, RT-PCR analysis was performed prior to whole-genome expression profiling, targeting these genes that are normally expressed during sporulation. The RT-PCR results revealed that the expression levels of the selected germination genes in mDS were significantly upregulated during sporulation (4 to 12 log_2_-fold, Fig 2A). These genes were not expressed during exponential growth (T = 2.5 h) but specifically expressed during *C*. *perfringens* sporulation (the following time points, see Fig 1). When *C*. *perfringens* was cultured in TGY broth (3% tryptic soy broth, 2% glucose, 1% yeast extract, 0.1% L-cysteine), the viable counts increased from 10^3^ CFU/ml at 3.0 h to 10^9^ CFU/ml at 6.0 h, but sporulation did not occur, as confirmed microscopically and by plating after heating for 10 min at 75°C (data not shown). The selected germination genes were not expressed in this medium as determined by RT-PCR (data not shown), confirming that their expression is specifically associated with *C*. *perfringens* sporulation. The expression patterns in time of the assessed genes in mDS showed similar patterns for most target genes; however, the onset of the gene up-regulation was later in the case of *prkC* (CPR_1709) and CPR_2348 than for the other target genes, namely around 1.5 h and 3 h later, respectively.

Subsequently, genome-wide transcriptional gene expression profiling was performed using custom microarrays. The expression data of the above mentioned germination genes were extracted from the dataset of the time-course microarray experiment and showed that their expression levels were increased one or more log_2_-fold during sporulation (Fig 2B). The data obtained using RT-PCR and microarray analysis followed similar expression patterns in time for the different genes, albeit that the log expression rates were lower for the microarray-based analyses than for the RT-PCR (Fig 2A and 2B).

### Identification of genes (putatively) involved in sporulation based on expression profiles and their occurrence in other sporeforming bacteria

**Fig 4:**
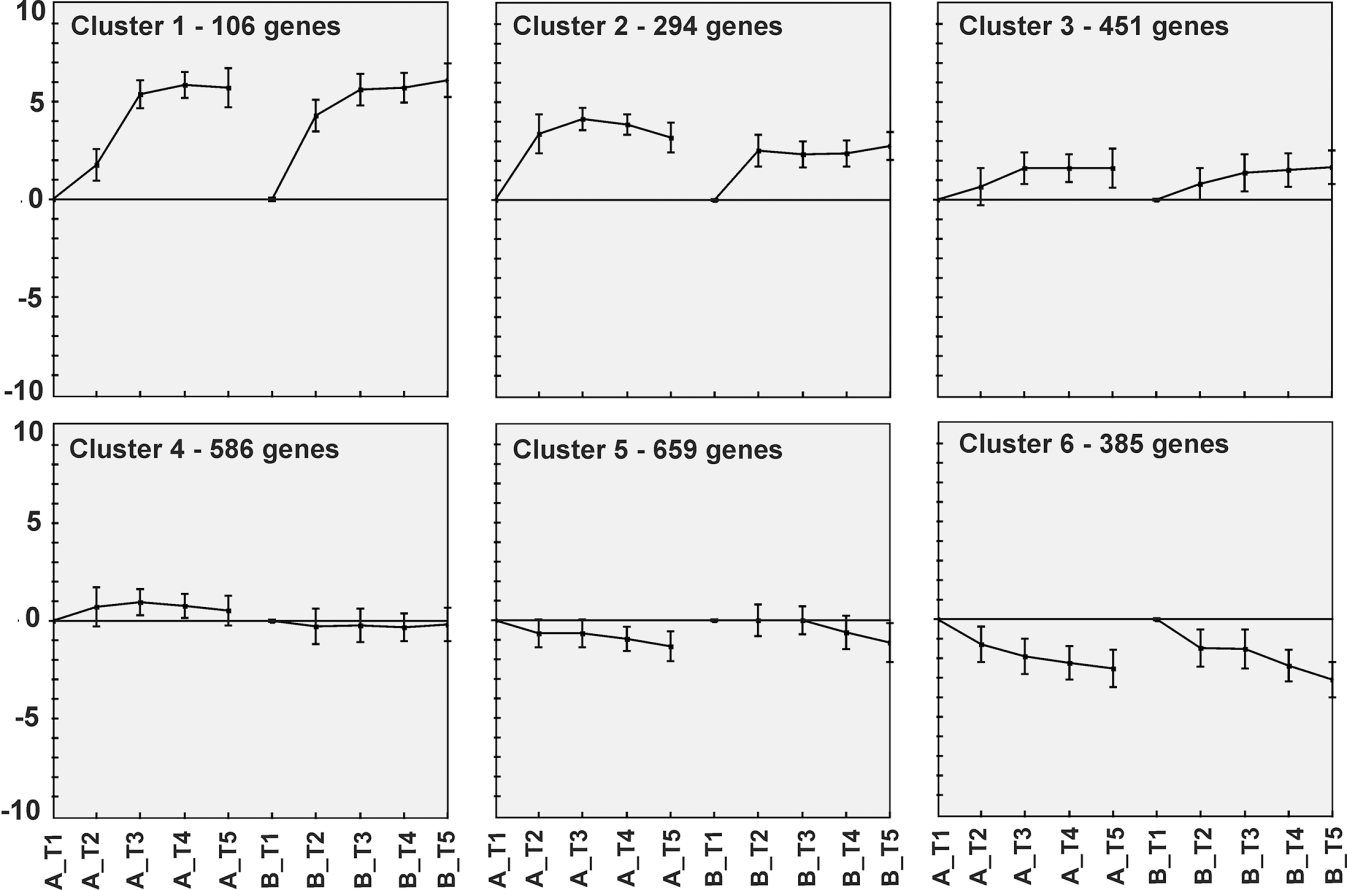
Results of *k*-means clustering of whole-genome expression profiles. Six clusters were generated based on comparison of point-wise similarity of genome-wide gene expression profiles during the sporulation time course with Euclidean distance (Genesis v 1.7.6). The samples collected at representative time points (T_1_-T_5_) of independent experiment duplicates (A and B) were equally weighted (see Methods and Materials). The data points show the average log_2_-transformed expression compared to exponential phase (T_1_) of all cluster members, with standard deviation indicated by the error bars.

*k*-means clustering of gene expression profiles during the *C*. *perfringens* sporulation process—based on point-wise similarity with equal weight—yielded six clusters of temporal expression. These clusters are presented in Fig 4 for the two independent experiments A and B. Many genes were up-regulated during sporulation to different extents: the expression levels of 106 genes in Cluster 1 exceeded 5 log_2_-fold increase. A total of 294 genes belonged to Cluster 2 and showed levels of up-regulation between 3 and 5 log_2_-fold (Fig 4). Lastly, Cluster 3 also contained genes that were up-regulated during sporulation (total 451genes), but at a lower level (around 2 log_2_-fold and maximally 3 log_2_-fold; Fig 4).

Known germination-related genes of *C*. *perfringens*—tested earlier using RT-PCR-were mainly found in Cluster 1 (*gpr*, *cspB*, *sleC*) and Cluster 2 (*gerKA*, *gerKC*, *gerKB*, CPR_2348), while *prkC* and *sleM* were found in Cluster 3. This showed that members of Clusters 1 and 2 may be associated with genes that are expressed during *C*. *perfringens* sporulation.

In a recent review, Galperin and colleagues [8] summarized the genes involved in sporulation, based on results of experimental functionality studies on *B*. *subtilis*, comparative genomics studies on sporeforming bacteria and proteomics studies on *C*. *difficile* spores [8,50]. *C*. *perfringens* homologs of known sporulation genes which were found to be up-regulated more than 3 log_2_-fold during sporulation of *C*. *perfringens* (belonging to Clusters 1 and 2) are shown in Table 2, listed per functional category. Additionally, S2 Table lists all *C*. *perfringens* homologs of (putative) sporulation genes that were summarized in the publication of Galperin and colleagues [8]. For each *C*. *perfringens* homolog of a candidate sporulation gene, the expression of these genes during sporulation is visualized. The expression levels of the *C*. *perfringens* homologs varied, leading to their distribution over the 6 clusters (see S2 Table).

**Table 2:** Cluster 1 and 2 *C*. *perfringens* homologs of known sporulation genes summarized by Galperin and colleagues [8]^*^.

Function category	Cluster 1	Cluster 2
**General sporulation**	CPR_1738 (*sigK*), CPR_1782 (*spoIVB*)**, CPR_2019 (*spoVAC*), CPR_2018 (*spoVAD*), CPR_2017 (*spoVAE*), CPR_2491 (*spoVT*).	CPR_2020 (*sigF*), CPR_1732 (*sigG*), CPR_2542 (*spmA*), CPR_2541 (*spmB*), CPR_2022 (*spoIIAA*), CPR_2021 (*spoIIAB*), CPR_2158 (*spoIID*), CPR_2475 (*spoIIE*), CPR_1778 (*spoIIM*), CPR_2012 (*spoIIP*), CPR_2157 (“*spoIIQ*”), CPR_1801 (*spoIIIAA*), CPR_1800 (*spoIIIAB*), CPR_1799 (*spoIIIAC*), CPR_1798 (*spoIIIAD*), CPR_1797 (*spoIIIAE*), CPR_1796 (*spoIIIAF*), CPR_1795 (*spoIIIAG*), CPR_1794 (*spoIIIAH*), CPR_2156 (*spoIIID*), CPR_1724 (*spoIVA*), CPR_2101 (*spoIVFB*), CPR_1053 (*spoVAF/GerA*), CPR_2490 (*spoVB*),CPR_1619 *(spoVB*), CPR_0528 (*spoVD*), CPR_1332 (*spoVR*)
**SASPs**	CPR_1411/CPR_2035 (*ssp*)	CPR_1870 (*ssp4*)
**Spore cortex**	CPR_2486 (“*yabP*”), CPR_2566 (*sleC*)	CPR_1699 (“*ylbJ*”), CPR_1993 (“*ygfC*”), CPR_1992 (“*yqfD*”) CPR_2361 (*cwlD*), CPR_2348
**Spore coat**	CPR_0933 (*cotJB*), CPR_0934 (*cotJC*), CPR_2191 (“*yabG*”), CPR_1593 (“*yybI*”)	CPR_2192 (*cotS*), CPR_1397 (“*yhbB*”)
**Spore coat maturation**		CPR_1770 (*dacB*)
**Spore germination**	CPR_2567 (*cspB*), CPR_2070 (“*cspA*”), CPR_2071 (“*cspC*”), CPR_2013 (*gpr*)	CPR_0615 (*gerKA*), CPR_0614 (*gerKB*), CPR_0616 (*gerKC*)
**Signaling**		CPR_2131 (“*ykuL*”), CPR_1334 (*prkA*)
**House cleaning**		CPR_0840 (*rbr*)
**Cell division, DNA replication**		CPR_1383 (*lonB*)
**Transport**		CPR_0437 (“*ytrB*”), CPR_2026 (“*yloB*”), CPR_2256 (“*oppC*”), CPR_2255 (“*oppD*”), CPR_2254 (“*oppF*”) CPR_2662 (“*ytvI*”)
**Cell wall metabolism**	CPR_1775 (*dacF*), CPR_1836 (*pdaA*) CPR_2453 (“*ykfA*”)	CPR_1878 (“*ybaN*”)
**Poorly characterized (R COGs)**		CPR_0756 (*hmp*)
**Uncharacterized (S COGs)**	CPR_1859 (*ytxC*), CPR_0147 (CD1511), CPR_1448 (CD3032), CPR_2589 (CD3613)	CPR_1348 (*ydfR/ydfS*), CPR_2517(*yerB*), CPR_1333 (*yhbH*), CPR_1731 (*ymxH*), CPR_1929 (*yunB*), CPR_1626 (*yuzA*), CPR2663 (*yyaC*), CPR2565 (*yyaD/ykvI*), CPR_0309 (CD0546), CPR_1322 (CD1594), CPR_2328 (CD2809), CPR_2525(CD3522), CPR_2297 (CD3580)

* Homologs of the known sporulation genes summarized in reference [8] but that do not have specific sporulation-associated increased expression (grouped in Clusters 3–6) can be retrieved from S1 Table.

** Locus tags of *C*. *perfringens* homologs are shown with their assigned *C*. *perfringens* gene names, gene names of the *Bacillus* genes are bracketed, and locus tags of *C*. *difficile* 630 are preceded by ‘CD’.

The majority of the genes with increased expression during sporulation (Clusters 1 and 2) belong to the functional categories “general sporulation”, SASPs, spore cortex, spore germination and transport. Other genes in these clusters had homologs that belong to functional categories such as housekeeping, cell division/DNA replication and cell wall metabolism (Table 2). The current study established the actual expression of a range of genes in *C*. *perfringens* during sporulation, including homologs of genes with established roles in sporulation in other species. This is a strong indication that these genes in *C*. *perfringens* have a functional role in sporulation and/or determine spore properties of this organism.

### Putative new sporulation and germination genes

Genes with the highest level of up-regulation during sporulation, namely those belonging to Cluster 1 (more than 5 log_2_-fold) and Cluster 2 (3–5 log_2_-fold), were subjected to further analysis. A particular focus was on genes that showed significantly increased expression during sporulation, but showed no homologs in the list drawn up by Galperin and colleagues [8].

Firstly, an inventory was made to establish the occurrence of the homologs of the Cluster 1 genes in the genomes of publicly available sporeforming Firmicutes. Cluster 1 contains 106 genes, including 25 genes that show homology with genes on the list published by Galperin and colleagues [8] (see Table 2). The analysis of the occurrence of homologs of the Cluster 1 genes in publicly available sporeforming Firmicutes revealed that 49 of 106 genes in the Cluster 1 were conserved amongst these strains. Within these conserved genes, 32 genes were not included in the list published by Galperin and colleagues [8] and they potentially play roles in mechanisms involved in sporulation and may determine spore properties. In addition, 33 genes were *Clostridium*-specific and the remaining 24 genes were *C*. *perfringens*-specific (Fig 3).

More *C*. *perfringens* homologs of known sporulation genes were found in Cluster 2, namely 64 on a total of 294 (see Table 2). All of these 294 genes were also subjected to a homology search in sporeforming Firmicutes. The presence/absence of the 294 gene homologs that belong to Cluster 2 in other Firmicutes is shown in S2 Table. Approximately 50% of the Cluster 2 genes, including 48 known genes, were highly conserved amongst sporeforming Firmicutes, while approximately 30% of the genes were *Clostridium*-specific and the remaining 20% *C*. *perfringens*-specific.

Our study established that for a number of likely candidate genes involved in spore formation expression indeed occurred in *C*. *perfringens* (highlighted in Fig 3). These included CPR_2486, a homolog of *B*. *subtilis yabP* (involved in spore coat assembly) [51]; CPR_1593, a homolog of *yybI* (encoding a protein located at the inner coat of spores) [52], CPR_2191, a homolog of *yabG* (encoding a protease involved in modification of spore coat proteins) [53], and CPR_2044, a homolog of *ytaF* which is an essential membrane protein in sporulation [54]. However, besides the enterotoxin-coding gene *cpe*, the majority of *Clostridium*-specific and *C*. *perfringens* specific genes are still poorly characterized or uncharacterized.

## Discussion

In this study, the succession of events during growth and the sporulation process of *C*. *perfringens* SM101 was characterized in mDS medium in detail. Representative time points for different phases throughout this process were selected and whole-genome expression profiling was performed throughout sporulation. This confirmed expression of known sporulation genes, of genes that had been predicted to play a role in sporulation in other studies, but also of genes that have so far not been associated with sporulation, but which may play a role in sporulation and potentially determine spore properties, including spore germination behavior.

*C*. *perfringens* strain SM101 showed fast exponential growth in mDS at 37°C with generation times of 11 min. Subsequent cell elongation and formation of phase bright, heat resistant spores took place within six hours after inoculation. Similar growth rates and time to sporulation have been reported for its parental food isolate NCTC8798 [55]. In previous transcriptomics studies on clostridial sporulation, determination of different sporulation phases was often based on optical density measurements, representing biomass accumulation [6,56]. The combined analyses of optical densities, total viable counts, heat resistant spore counts, and microscopic imaging in this study showed a discrepancy between the increase of the OD_600_ and the total viable counts due to cell elongation. In addition, it was evident that only ~1% of the total number of cells produced mature, heat resistant spores. This percentage is low given the microscopic observation that a high proportion of vegetative cells produced phase bright spores (see microscopic image at T4 in Fig 1). A possible explanation for this phenomenon is that immature spores were actively lysed and provided essential nutrients for a subpopulation to complete the formation of resistant spores. Similar observations have recently been reported during *B*. *subtilis* sporulation, with a role for the *skf* and *sdp* operons, encoding spore killing factor and the cannibalism toxin SDP, respectively [57–60]. *Clostridium* “cannibalism” during sporulation has not been reported so far, and no *skf* or *sdp* homologs were found in the *C*. *perfringens* SM101 genome (data not shown).

In recent years, a substantial number of available genome sequences of sporeforming bacteria have become available. This has allowed for comparative genomics studies to establish similarities and differences in the sporulation process and germination properties of relatively well-studied *Bacillus* species (particularly *B*. *subtilis*) and *Clostridium* species. The predicted presence of (putative) orthologs of known *Bacillus* sporulation genes in *Clostridium* species suggests that the sporulation cascades of these two genera overlap at least in part with a core sporulation genome that has been reported [8]. However, there are also clear differences [7,24,61]. One of the hallmark differences in sporulation is that the initiation of this process in bacilli is mediated by phosphorylation of Spo0A through a phosphorylation cascade [62], while in clostridia, Spo0A is believed to be phosphorylated directly [63]. In addition, bacilli and clostridia may possess different germination mechanisms [24,64].

In this study, the expression during sporulation of known *C*. *perfringens* germination genes with experimentally established roles was confirmed, namely *gerKA*, *gerKC* [21], *gerKB* [19], *sleC* [27], *cspB* [26] and *sleM* [30]. In addition, the actual expression of a large number of so far merely predicted sporulation and germination genes was confirmed in *C*. *perfringens* in this study. This included most of the genes that were listed by Galperin and colleagues [8] which are deemed essential for sporulation, spore properties and germination. A substantial number of orthologs of genes that were listed in their study [8] showed high upregulation, including 25 genes in Cluster 1 and 64 in Cluster 2 (see Table 2).

Furthermore, it was found that a range of other genes were upregulated during sporulation of *C*. *perfringens* that were also highly conserved in other Firmicutes, pointing to so far uncharacterized genes that play a role in sporulation and/or determine spore properties.

For the *C*. *perfringens* genes with the highest levels of up-regulation during sporulation (*i*.*e*. Cluster 1 members) a search was performed for homologs in sporeforming Firmicutes (presented in Fig 3). The genes were grouped according to their conservation levels and their functional classes. Around half of the genes belonging to Cluster 1 were found to be conserved in sporeforming Firmicutes. Cluster 1 also includes genes that encode predicted proteins that are involved in amino acid/ferrous ion transport and enzymatic modifications of functional groups (transferases), which have previously not been associated with the sporulation process in particular. These genes putatively encode proteins associated with energy generation, e.g. oxidoreductase (CPR_1769), flavodoxin (CPR_0519), ferrous iron transport (operon CPR_0970-CPR_0971-CPR_0972), aminotransferases (operon CPR_0567-CPR_0568- CPR_0569) and carbonate polymerization (CPR_2159). These findings strongly suggest that these mechanisms are associated with sporulation, and the genes potentially have an influence on properties of the spores that are formed. The other half of the genes in Cluster 1 had homologs only in clostridia, or even more particularly, only in *C*. *perfringens*. The functions of most *Clostridium*-specific and *C*. *perfringens*-specific genes in Cluster 1 remain uncharacterized, except for the *cpe* gene, encoding the enterotoxin.

A similar analysis was performed on the genes belonging to Cluster 2, which showed less pronounced—yet significant—upregulation during sporulation. Cluster 2 contained 64 homologs of known sporulation genes out of 294 in total (see Table 2). In addition, 48 genes were expressed in *C*. *perfringens* during sporulation and showed orthologs in most other sporulating Firmicutes. These genes are also important candidates and may play a role in sporulation of *Bacillus* and *Clostridium* spp., such as CPR_0449 (annotated as a sensor histidine kinase, may potentially play a role in sensing environmental signal) and CPR_0558 (belongs to Mg^2+^ transport ATPase MgtC family).

Another 75 genes with orthology to genes listed in [8] showed no significant up-regulation or levels of up-regulation that were below 3 log_2_-fold, and therefore belonged to Cluster 3, 4, 5 or 6 (S1 Table) despite having established roles in sporulation in other species. Potential explanations for such observations could include the following: the level of up-regulation of genes involved in initiation of sporulation or early stage sporulation may be underestimated as the time point of sampling is close to the reference time point (T_1_) of exponential growth; sporulation is tightly regulated and certain genes have inhibiting effects on sporulation genes, e.g. genes encoding anti-transcriptional factors (e.g. *spoIIAA*); genes are expressed during sporulation but the required levels of transcripts are below 3 log_2_-fold (*e*.*g*. genes encoding sigma-factors); pseudogenes may be present that have high sequence similarity with known sporulation genes but their expression levels were very low or completely silenced throughout the sporulation process. Nevertheless, we demonstrated that 89 genes listed in [8] not only have orthologs in *C*. *perfringens*, but are actually expressed during sporulation of this pathogen, indicative of a functional role of these orthologs.

The current study has provided a genomic insight into expression patterns during sporulation of *C*. *perfringens* with a specific focus on sporulation and germination genes, thereby confirming expression of known and predicted genes with a role in these processes. Moreover, the study has identified expression of genes during late stage sporulation of this bacterium that are highly conserved in other Firmicutes and may play so far unidentified roles in sporulation and determine spore properties, including germination behavior.

## Supporting Information

S1 Fig Hybridization scheme of the microarray experiment. Whole-genome RNA expression of two separate *C*. *perfringens* sporulating cultures (A and B) was compared at five representative time points (T_1_ to T_5_). Each of the five time points corresponded with a specific sporulation phase described in the Results section. cDNA samples of the different time point and of the different cultures (A and B) were coupled with fluorescent dye Cy3 and Cy5 in two different batches. The hybridization scheme onto 13 microarrays is presented.
(TIF)

S1 Table Homologs of (putative) sporulation genes and their expression during *C*. *perfringens* sporulation. Sporulation genes functionally summarized and categorized by Galperin and colleagues [8] are listed in the 1^st^ major column. The resulting *C*. *perfringens* homologs are listed in the 2^nd^ major column. Their expression patterns and cluster numbers resulted from *k*-means clustering are shown in 3^rd^ major column.
(XLSX)

S2 Table *C*. *perfringens* genes in Cluster 2 and distribution of their homologs in other sporeforming Firmicutes. The genes with known sporulation-related function are indicated in bold against a grey background; the ones with putative functions (poorly characterized and uncharacterized) have a grey background only while the others without specific highlights have not been associated with sporulation or are not known to determine spore properties.
(XLSX)
