# Supplementary figures and images for: Genome-Wide Transcriptional Profiling of Clostridium perfringens SM101 during Sporulation Extends the Core of Putative Sporulation Genes and Genes Determining Spore Properties and Germination Characteristics
Source: PLoS One. 2015 May 15;10(5):e0127036. doi: 10.1371/journal.pone.0127036 (PMC4433262; doi:10.1371/journal.pone.0127036)

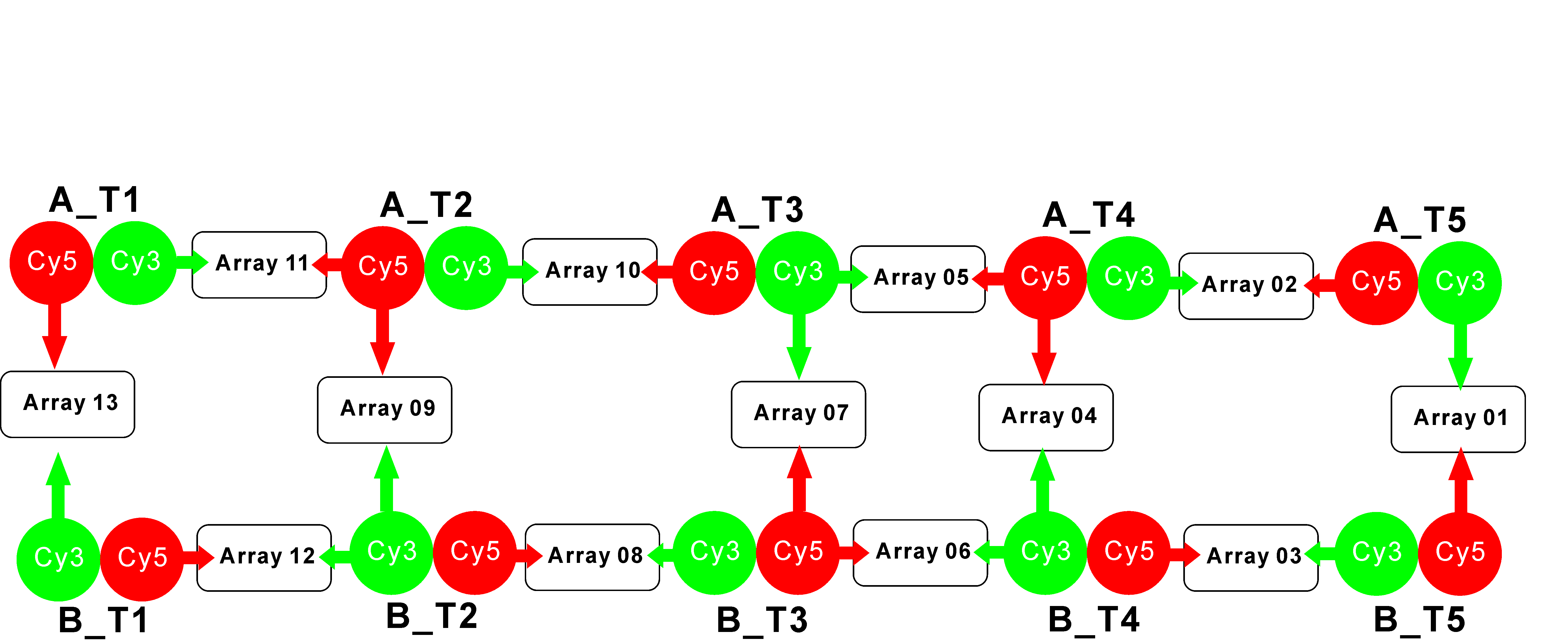

Supplement: S1 Fig — Whole-genome RNA expression of two separate C. perfringens sporulating cultures (A and B) was compared at five representative time points (T1 to T5). Each of the five time points corresponded with a specific sporulation phase described in the Results section. cDNA samples of the different time point and of the different cultures (A and B) were coupled with fluorescent dye Cy3 and Cy5 in two different batches. The hybridization scheme onto 13 microarrays is presented. (TIF) [file pone.0127036.s001.tif]
